# A Liquid Band-Aid with Mesenchymal Stem Cell-derived Exosomes for Wound Healing in Mice

**DOI:** 10.2174/0113892010331302240913114112

**Published:** 2024-09-30

**Authors:** Wei-Yuan Zhang, Tian-Jiao Meng, Jia Hu, Li Wen, Li Du, Xiao-Chen Cheng, Li-Sheng Wang, Feng-Jun Xiao, Yu-Xin Lu

**Affiliations:** 1 Department of Special Medicine, School of Basic Medicine, Qingdao University, Qingdao, 266071, PR China;; 2 Laboratory of Molecular Diagnosis and Regenerative Medicine, the Affiliated Hospital of Qingdao University, Qingdao 266000, PR China;; 3 Beijing Radiation Center, Beijing Institute of Radiation Medicine, Beijing, 100850, PR China;; 4 Department of Cardiovascular, the Sixth Medical Center of Chinese PLA General Hospital, Beijing 100853, China;; 5 The Second Hospital of Jilin University, School of Nursing, Jilin University, Changchun, Jilin, 130021, PR China

**Keywords:** Human umbilical cord, mesenchymal stem cells, exosome, chitosan, liquid band-aid, wound repair, biomaterial

## Abstract

**Introduction/Objective:**

This study aimed to examine the effect of a human umbilical cord mesenchymal stem cell-derived exosome (hUC-MSC-Exo) liquid band-aid on wound healing in mice.

**Methods:**

hUC-MSC-Exos were prepared from the supernatant *via* ion exchange chromatography. The composition ratio of the chitosan liquid band-aid was optimized to form a film and encapsulate hUC-MSC-Exo. The biological effects of chitosan exosome liquid band-aid on human umbilical vein endothelial cells (HUVECs) were observed, and its anti-bacterial properties were tested. BALB/c mice with back skin injury were randomly divided into chitosan exosome liquid band-aid group (CS-Exo), chitosan liquid band-aid group (CS), and normal saline control group (Con), and wound healing was evaluated post-treatment. Skin tissue samples post-treatment were collected for H&E staining.

**Results:**

The hUC-MSC-Exo was prepared and characterized. The optimum conditions for film formation were 1% chitosan solution and 15% poloxamer 407/poloxamer 188 (pH 5.0 ~ 7.0). The chitosan exosome liquid band-aid promoted HUVEC proliferation and migration and markedly inhibited *Escherichia coli* and *Staphylococcus aureus* growth *in vitro*. *In vivo*, the wound healing rate in the CS-Exo group was higher than that in the Con and CS groups. Fourteen days post-treatment, the wounds completely healed, and hair grew normally, which was consistent with H&E results. Mouse weights in each group did not change significantly after administration, indicating that the chitosan exosome liquid band-aid had no obvious toxic side effects.

**Conclusion:**

Local chitosan exosome liquid band-aid application can promote wound healing in mice, and the mechanism could be related to hUC-MSC-Exo-induced vascular endothelial cell proliferation and migration.

## 1 INTRODUCTION

The skin is the largest external organ of the human body and is in direct contact with the external environment [1]. Accordingly, it is vulnerable to various injuries due to mechanical, physical, chemical, and biological factors and, as a result, can be subjected to trauma [2]. Skin wounds usually heal spontaneously. However, severe lesions can result in wound-healing difficulties [3]. Therefore, the development of approaches that accelerate wound healing is important for skin wound repair [4]. Recently, Mesenchymal Stem Cells (MSCs), a major stem cell type used in regenerative medicine, have been applied for skin wound healing [5]. MSCs have regenerative activity during tissue repair through anti-inflammatory, anti-apoptotic, and angiogenesis-promoting properties, in addition to regulatory effects on cell proliferation, differentiation, and migration [6-8]. Exosomes, extracellular vesicles generated by cells, carry various biological molecules, such as miRNAs, proteins, lipids, and metabolites. Moreover, they exhibit different biological activities and can be used for cell-free therapy [7]. MSC-derived exosomes have activities similar to those of their original cells [9], and multiple studies have confirmed that they can promote wound repair and skin regeneration [9, 10]. Large-scale preparation procedures have also been optimized and applied for the production of exosome products [11]. However, exosomes in a liquid state do not easily attach to local skin wounds due to their mobility.

Biomaterials can prolong the retention time of exosomes on wound surfaces, helping to maintain their biological activity. The development of hydrogel dressings with adhesive ability is a promising method for treating skin wound healing [12, 13]. The adhesion methods of hydrogels are generally divided into chemical and physical kinds, most of which rely on the transition from a liquid state to a solid state to achieve adhesion [14]. Chemical adhesion achieves stable adhesion to damaged tissues through covalent bonds, but its clinical application is currently limited due to its potential toxicity [15]. Physical adhesion is usually achieved through mechanisms, such as hydrogen bonding and electrostatic force, providing fast and strong adhesion [12]. Among the various biological materials that maintain the structure through the action of surface tension and intermolecular forces to achieve physical adhesion to produce hydrogels, poloxamer stands out for its changeable gelatinization properties [16]. As a low-flow liquid with thermally reversible properties, the fluid state of poloxamer at room temperature facilitates drug delivery, while the gel state at body temperature above the sol-gel transition temperature promotes prolonged release of pharmacological reagents [16]. Common liquid suppository substrates, poloxamer 407 and 188, in appropriate proportions, can be used as a membrane-forming biomaterial, liquid band-aid, to rapidly form a tight adhesive membrane structure [17].

In order to form adhesive films in skin wounds, polymers with viscoelastic properties or *in situ* film-forming properties must be used to assist film formation. Chitosan is a natural polymer that can form film *in situ* [18]. Due to its excellent adhesion properties, antibacterial and antioxidant activities, and degradability, chitosan has been proven to be an effective drug carrier for wound healing treatment and has been widely used in medical dressings [19]. In this study, exosome-derived stem cells were prepared and combined with a liquid band-aid to prepare a chitosan exosome liquid band-aid. In addition, the pro-healing effect of this preparation was verified through its local application to mouse wounds, suggesting a novel approach and basis for wound repair research.

## 2 MATERIALS AND METHODS

### 2.1 Materials

The materials included human MSC culture medium (Beijing Kelin Biotechnology Co., Ltd.), mesenchymal stem cell differentiation medium (ScienCell, USA), anti-CD90-FITC, anti-CD14-PE, anti-CD34-PE, anti-CD45-FITC, anti-CD105-APC, and anti-CD73-APC (eBioscience, USA), anti-CD63 and anti-TSG101 antibodies (Abcam, UK), and anti-HSP70 antibody (Proteintech, USA), water-soluble chitosan (Beijing Coolaibo Technology Co., Ltd.), and poloxamer 188, poloxamer 407, and aminoacetic acid (Beijing Solaibao Technology Co., Ltd.).

### 2.2 Animals

Forty-five BALB/c mice (male, SPF grade, 6–7 weeks of age, 20–22 g) were purchased from Beijing SIPEIFU Biotechnology Co., Ltd. (laboratory animal license number: SCXK [Beijing] 2019-0010).

### 2.3 Culture and Identification of Human Umbilical Cord MSCs (hUC-MSCs)

Primary UC-MSCs were obtained from Allcare Biotechnology Co., Ltd. hUC-MSCs at passage 4 (P4) were observed under a microscope and photographed. The expressions of the positive markers CD73, CD90, and CD105 and the negative markers CD14, CD34, and CD45 on the surface of hUC-MSCs were detected *via* flow cytometry. P5 hUC-MSCs were then induced to undergo osteogenic and adipogenic differentiation.

### 2.4 Preparation of hUC-MSC Exosomes (hUC-MSC-Exos)

The culture supernatant of P6–P8 hUC-MSCs was collected and filtered through a 0.45 μm filter membrane, and the hUC-MSC-Exos were prepared using ion-exchange chromatography. The Source Q30 adsorption column was first equilibrated using loading buffer (100 mM Tris-HCl, 10 mM EDTA, 0.4 M NaCl, pH=7.5). The hUC-MSC culture medium was then passed through a Source Q30 column at a flow rate of 15 mL/min to adsorb hUC-MSC-Exos. Elution buffer (100 mM Tris-HCl, 10 mM EDTA, 1 M NaCl, pH 7.5) was used to elute hUC-MSC-Exos. The collection of the exosome eluate started at 770 mL and was stopped at 831 mL; the detection wavelength was 260 nm.

### 2.5 Transmission Electron Microscopy

The prepared exosomes were dropped, in a volume of 15 μL, on the electron microscope copper net, and 4% paraformaldehyde was added for fixation. Then, uranyl acetate was added to stain the exosomes. The excess liquid was absorbed with filter paper, and exosome morphology was observed and photographed using a transmission electron microscope (Hitachi, Japan).

### 2.6 Nanoparticle Tracking Analysis (NTA)

The prepared exosomes were diluted with normal saline at a ratio of 1:5000 to 1:20000 and loaded for NTA (ZetaView, Germany) using a syringe. The data were recorded, and the analysis report was saved.

### 2.7 Western Blotting

The protein concentration of the prepared hUC-MSC-Exos was determined using a BCA protein concentration assay kit (Thermo, USA). RIPA was added to the exosomes, which were mixed by pipetting on ice; then, SDS loading buffer was added, and the sample was incubated at 100°C for 5 min. Exosome proteins (5 μg) were electrophoresed, separated on 4–20% SDS-PAGE gradient gels (Bio-Rad, Hercules, CA, USA), and transferred to PVDF membranes. After blocking, the membranes were incubated with rabbit anti-human CD63, anti-TSG101, and anti-HSP70 primary antibodies dilutants overnight at 4°C. The membrane was then incubated with goat anti-rabbit fluorescent secondary antibody for 1 h, visualized, and photographed using a chemiluminescence kit (Merck Millipore, Billerica, MA, USA).

### 2.8 Preparation of Chitosan Exosome Liquid Band-aid

Water-soluble chitosan was dissolved in purified water to prepare chitosan solutions of different concentrations (0.5%, 1%, 2%, and 3%), which were then subjected to defoaming. Subsequently, 0.1% aminoacetic acid was added and dissolved by stirring. Poloxamer 407 and poloxamer 188 in different proportions (10%, 15%, 20%, 25%, 30%) were added to prepare solutions with different concentrations, which were incubated overnight at 4°C. Poloxamer 407 and poloxamer 188 were completely dissolved, and a chitosan liquid band-aid was prepared. hUC-MSC-Exos were added, and the sample was mixed and filtered through a 40 μm filter to prepare the chitosan exosome liquid band-aid, which was stored at 4°C.

### 2.9 Determination of chitosan exosome liquid band-aid membrane formation

Water soluble chitosan was used to prepare 0.5%, 1.0%, 2.0%, and 3.0% chitosan solutions. For different concentrations of chitosan solutions, 10%, 15%, 20%, 25%, and 30% poloxamer 407 / poloxamer 188 and 0.1% aminoacetic acid were added to prepare the liquid band-aid. Three drops of the liquid band-aid, at different concentrations, were then evenly applied to the skin of the shaved mice on the backs. The smoothness of the band-aid was observed after film formation, and the film formation time was recorded. All animal experiments were approved by the Laboratory Animal Centre of the Academy of Military Medical Sciences.

### 2.10 Culture of human umbilical vein endothelial cells (HUVECs) and chitosan exosome liquid band-aid treatment

HUVECs were cultured in HUVEC-specific medium (Pricella, China). Cells were seeded at 1×10^5^ cells/cm^2^ in wells of cell culture plates, and normal saline, chitosan, or chitosan exosome liquid band-aid was added to the co-culture. The cells were collected at different time points for testing.

### 2.11 Giemsa Staining

After co-culturing the HUVECs with normal saline, chitosan, or the chitosan exosome liquid band-aid for 48 h, the medium was aspirated, and 4% paraformaldehyde was added to fix the cell morphology. HUVECs were stained using a Giemsa staining kit (Solarbio, China), and cell morphology and numbers were determined under an optical microscope, with photographs taken for preservation.

### 2.12 CCK-8

HUVECs were co-cultured with normal saline, chitosan, or the chitosan exosome liquid band-aid for 0, 24, 48, 72, or 96 h, and CCK-8 reagent (Merck, USA) was added to the culture medium and incubated at 37°C for 2 h. Absorbance was measured at 450 nm using a microplate detector.

### 2.13 Scratch Tests

HUVECs were inoculated into and cultured in 6-well plates until the cell fusion was 100%. A straight line was drawn on monolayer cells with the tip of a sterile pipette, and the cells were rinsed three times with phosphate buffer solution to remove dead cells. Normal saline, chitosan, or chitosan exosome liquid band-aid was added to the scratched HUVECs, and cell migration into the scratch was observed and photographed under an optical microscope at 0, 6, and 12 h.

### 2.14 Antibacterial Test

Strains of *Escherichia coli* and *Staphylococcus aureus* were activated and expanded in the LB liquid medium, and the liquid concentration was measured. After the bacterial solution was evenly applied to the LB solid medium with a coating stick, the filter paper soaked with normal saline, chitosan, or the chitosan exosome liquid band-aid was placed on the filter paper. After 12 h, bacterial growth around the filter paper was observed and photographed.

### 2.15 Establishment and Treatment of Animal Skin Wound Model

After 5 days of adaptive feeding, 45 male BALB/c mice were randomly divided into three groups: the normal saline control group (Con group), chitosan liquid band-aid group (CS group), and chitosan exosome liquid band-aid group (CS-Exo group), with 15 mice in each group. The mice were anesthetized *via* an intraperitoneal injection of 1.0% sodium pentobarbital, their back hair was shaved, and their back skin was sterilized with 75% ethanol. The whole cortical skin was cut from the backs of the mice using a circular medical perforator with a diameter of 8 mm. After the injury, normal saline, the chitosan band-aid, or the chitosan exosome liquid band-aid was applied to the wound area, once per day for three consecutive days, at 100 μL per mouse. The mice were housed in cages (one mouse/cage) to avoid licking and rubbing the wounds of other animals. Wound healing was observed daily after administration. The wounds were photographed on days 0, 3, 7, and 14, and the body weights of the mice were recorded. Image analysis software (ImageJ) was used to measure the wound area and calculate the wound healing rate. The wound area on day 0 was considered the initial area, and the wound healing areas on days 3, 7, and 14 were calculated. The wound healing rate was determined by comparing the wound area with the wound area on day 0 (the wound area was less than 1 mm^2^, and the wound was considered healed). All animal experiments were approved by the Laboratory Animal Centre of the Academy of Military Medical Sciences.

### 2.16 Hematoxylin-eosin (H&E) Staining

On days 3, 7, and 14 after administration, five mice in each group were euthanized, the hair on the wound site on the backs of the mice was shaved, and the skin on the wound site was cut and fixed in 10% formalin solution. After 24 h, the tissues were embedded in paraffin, sliced, and stained with H&E. The cells were then observed under a light microscope and photographed.

### 2.17 Statistical Analysis

All values are presented as the mean ± the standard deviation. Student’s *t*-tests were used for comparisons between two groups, and an analysis of variance was used for comparisons among multiple groups. Differences between groups were considered significant at *p* < 0.05.

## 3 RESULTS

### 3.1 Culture and Identification of hUC-MSC

**Fig. (1):**
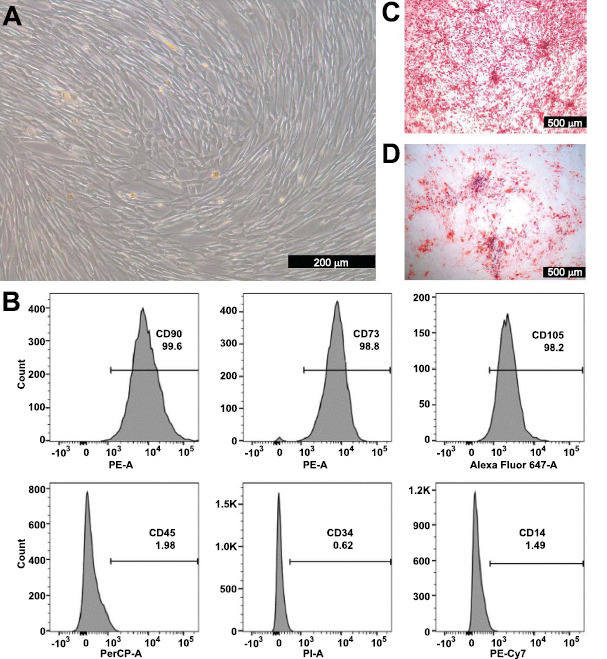
**Culture and identification of human umbilical cord mesenchymal stem cells (hUC-MSCs). A**) Morphological observation of hUC-MSCs. **B**) Flow cytometric analysis of CD73, CD90, CD105, CD14, CD34, and CD45 on the surface of hUC-MSCs. **C**) hUC-MSC osteogenic differentiation. **D**) hUC-MSC lipogenic differentiation.

The umbilical cord tissue was digested and cultured to obtain hUC-MSCs. The hUC-MSC growth resembled a vortex, and cells adhered to the vessel wall, exhibiting long spindle shapes consistent with the morphology of MSCs (Fig. **1A**). Regarding the hUC-MSC phenotype, measured *via* flow cytometry, the percentage of CD90, CD73, and CD105 expression was more than 95%, and that of CD45, CD34, and CD14 was less than 5% (Fig. **1B**). After 14 days of osteogenic induction, hUC-MSCs appeared red under the microscope, indicating matrix mineralization (Fig. **1C**). After 21 days of adipogenic induction, lipid droplets were present in hUC-MSCs, as observed using a microscope (Fig. **1D**). These characteristics confirmed the differentiation ability of hUC-MSCs, which is consistent with that in the literature [20]. These results indicate that the hUC-MSCs prepared in this study met international criteria.

### 3.2 Preparation and Identification of hUC-MSC-Exos

**Fig. (2):**
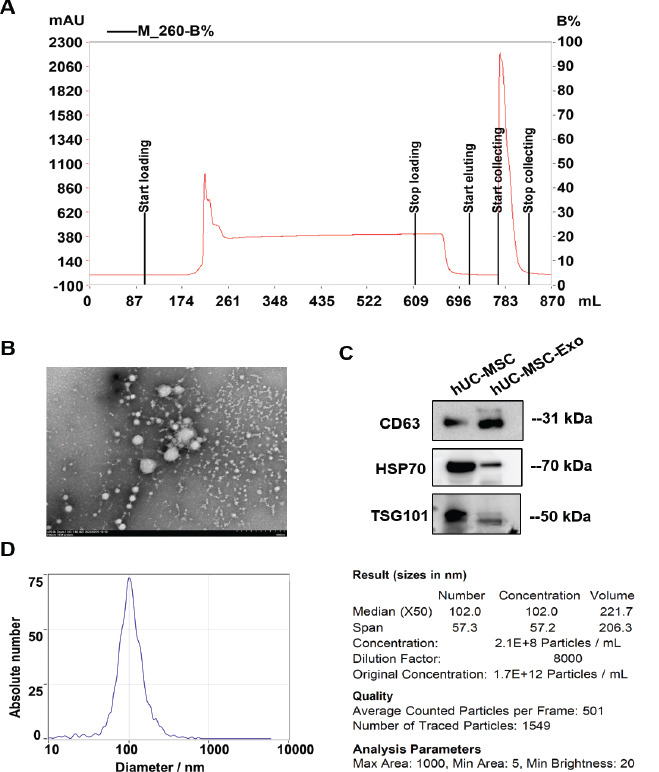
**Preparation and identification of human umbilical cord mesenchymal stem cell-derived exosomes (hUC-MSC-Exos). A**) Preparation process for hUC-MSC-Exos. **B**) hUC-MSC-Exos observed *via* transmission electron microscopy. **C**) Western blotting was used to detect the exosome-specific markers CD63, HSP70, and TSG101. **D**) Particle size distribution of hUC-MSC-Exos, as detected using a nanoparticle tracking analyzer.

hUC-MSCs were amplified *in vitro*, and the supernatant was collected and filtered at 0.45 μm to remove cell debris and cell vesicles. hUC-MSC-Exos were isolated and purified using ion-exchange chromatography (Fig. **2A**). hUC-MSC-Exos exhibited a standard dispersion and membrane structure under a transmission electron microscope (Fig. **2B**). Furthermore, western blotting showed that hUC-MSC-Exos expressed the exosome-specific markers, including CD63, HSP70, and TSG101 (Fig. **2C**). Particle size analysis showed that the peak size distribution of the exosome particles was 102 ± 57.2 nm, and the particle concentration was 1.7×10^12^ particles/mL (Fig. **2D**). This indicates that hUC-MSC-Exos were successfully extracted [21, 22], making them suitable for the further preparation of the liquid band-aid.

### 3.3 Chitosan Liquid Band-aid Membrane Formation

Based on the smoothness and film-formation speed of the liquid band-aid, the chitosan concentration and poloxamer 188 / poloxamer 407 ratio were optimized to generate a liquid band-aid. Moreover, appropriate preparation procedures for the liquid band-aid were screened and validated. Among them, the smoothness was divided into surface smoothness and smoothness after rubbing (1–5 points), and the film-formation speed was classified as excellent (14 min), good (4–6 min), or passable (>6 min). The results showed that the liquid band-aid composed of a 1% chitosan solution, 15% poloxamer 407 / poloxamer 188, and 0.1% aminoacetic acid (pH 5.0–7.0) had better smoothness and a shorter film formation period than the other groups (Table **1**). Therefore, this formulation was used to prepare the exosome liquid band-aid for subsequent animal experiments.

### 3.4 Chitosan Exosome Liquid Band-aid Promotes HUVEC Proliferation and Migration

**Fig. (3):**
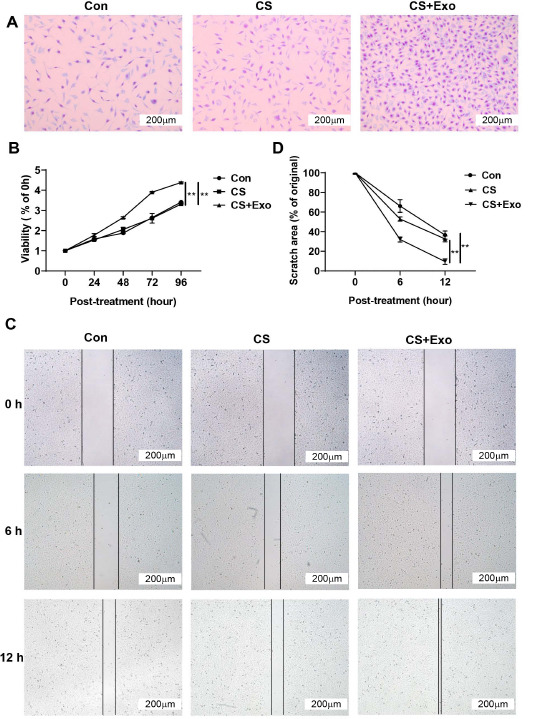
**Chitosan exosome liquid band-aid promotes human umbilical vein endothelial cell (HUVEC) proliferation and migration. A**) Giemsa staining of HUVECs after 48 h co-culture with normal saline (Con), the chitosan liquid band-aid (CS), and the chitosan exosome liquid band-aid (CS-Exo). **B**) Proliferation of HUVECs treated with normal saline, CS, or CS-Exos at 0, 24, 48, 72, and 96 h, as measured based on CCK-8 assays. **C**) Migration of HUVECs treated with normal saline, CS, or CS-Exo at 0, 6, and 12 h, as determined by performing a scratch test. **D**) Analysis of the scratch area in C.

After HUVECs were co-cultured with normal saline, the chitosan liquid band-aid, or the chitosan exosome liquid band-aid, cell proliferation was assessed by measuring the number of cells. The number of cells in the chitosan exosome liquid band-aid treatment group was increased significantly, and similarly, the proliferation index was significantly increased (Fig. **3A** and **B**). The chitosan exosome liquid band-aid also significantly improved the post-scratch migration ability of HUVECs, suggesting that it can promote wound healing *in vitro* (Fig. **3C** and **D**).

### 3.5 Bacteriostatic Effects of Chitosan Exosome Liquid Band-aid on *E. coli* and *S. aureus*

**Fig. (4):**
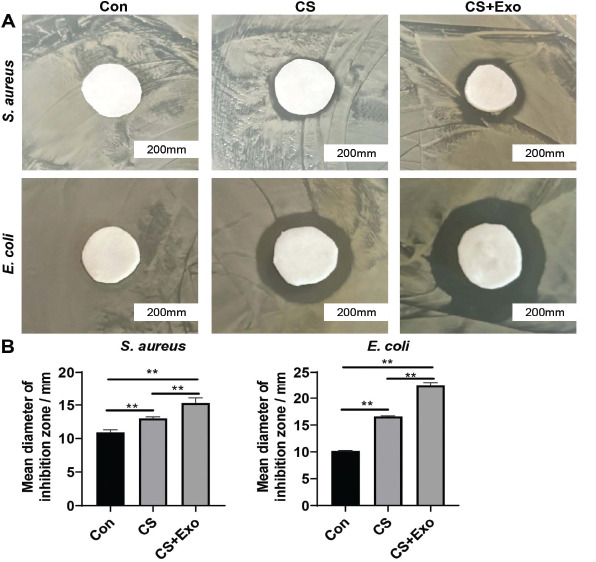
**Bacteriostatic effect of chitosan exosome liquid band-aid on *Escherichia coli* and *Staphylococcus aureus*. A**) Photographs taken of the inhibition zone induced by CS or CS-Exo. **B**) Analysis of the mean diameter inhibition zone in A.

Representative gram-negative bacteria (*E. coli*) and gram-positive bacteria (*S. aureus*) were selected to test the bacteriostatic properties of the chitosan liquid band-aid and chitosan exosome liquid band-aid. Twelve hours after inoculation, bacteriostatic zones were observed around the chitosan or chitosan exosome liquid band-aid and compared with those when using filter paper soaked in normal saline. The diameter of the bacteriostatic zone was largest with the chitosan exosome liquid band-aid, indicating that it had strong bacteriostatic effects (Fig. **4A** and **B**).

### 3.6 Chitosan Exosome Liquid Band-aid Promotes Wound Healing in Mice

**Fig. (5):**
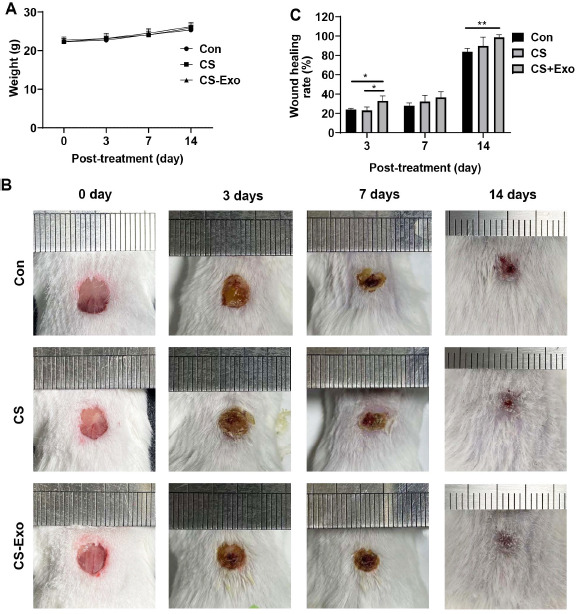
**Wound changes, wound healing rate, and body weights of mice after treatment with chitosan exosome liquid band-aid. A**) Mouse body weight changes. **B**) Skin injury changes on days 0, 3, 7, and 14. **C**) Wound healing rate in mice on days 0, 3, 7, and 14.

The mice in each group were weighed on days 0, 3, 7, and 14 after modeling and liquid band-aid application. There was no significant change in the body weights of the mice in each group (Fig. **5A**). This suggests that the chitosan exosome liquid band-aid had no significant toxic effects on the mice. Wound healing in each group was obviously reduced at day 3 post-treatment (Fig. **5B**). On day 7, the wound areas in the three groups were significantly reduced, and the scars formed in the CS-Exo group almost completely covered the wounds. Granulation tissue was observed at the center of the wound in the Con and CS groups, and the scar did not completely cover the wound. By day 14, the skin wounds in the CS-Exo group healed completely. Moreover, hair follicles had recovered, and hair grew in the center of the wound. A strong wound-healing effect was thus observed in the CS-Exo group.

Images of wound healing in mice were analyzed using image analysis software (ImageJ). The wound healing rate in the CS-Exo group was significantly higher than that in the Con group and CS group at 3 days (*p*<0.05; Fig. **5C**). Fourteen days after injury, the healing rate in the CS-Exo group was higher than that in the Con groups. Ultimately, the integrated data indicated that the chitosan exosome liquid band-aid could promote wound healing in mice.

**Fig. (6):**
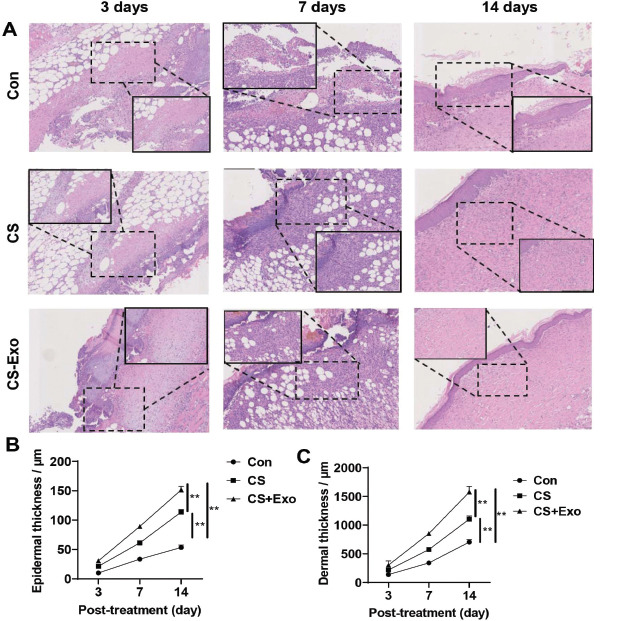
**Pathology of wounds in the control (Con), chitosan liquid band-aid (CS), and chitosan exosome liquid band-aid (CS-Exo) treatment groups. A**) Photographs taken for H&E staining. **B**) Statistical analysis of epidermal thickness. **C**) Statistical analysis of dermal thickness.

Pathological changes were also observed. Three days after injury, high levels of purulent secretions, exudate neutrophils, and tissue necrosis were observed in the wounds of the Con group. A small amount of subcutaneous granulation tissue was observed in the wounds of mice in the CS group. However, large amounts of subcutaneous granulation tissue and neovascularization were observed in wound tissues in the CS-Exo group. On day 7 post-treatment, compared with observations in the CS and Con groups, the necrotic area was reduced, acute inflammatory signs were alleviated, and fibroblasts were observed near the wound in the CS-Exo group. On day 14 post-treatment, the granulation tissue in the CS-Exo group had matured, whereas in the CS and Con groups, inflammatory infiltration and thicker epidermal tissue were still observed (Fig. **6A**). Changes in mean epidermal thickness and mean dermal thickness were measured. At 3, 7, and 14 days after injury, the epidermal thickness and dermal thickness in the CS+Exo group were significantly higher than those in the CS and Con groups (Fig. **6B**). Overall, enhanced pathological wound healing, relative to that in the Con and CS groups, was observed in the CS-Exo group.

## 4 DISCUSSION

Exosomes are extracellular vesicles that are actively secreted by cells, and they carry a variety of proteins, nucleic acids (*e.g*., miRNAs, mRNA, and lncRNAs), and other active substances that can affect the function of target cells and regulate their biological behaviors through specific binding to target cells [23]. In addition, active substances are released to facilitate material transport and signal transmission, in addition to participating in information exchange between cells and various pathophysiological processes [24]. MSC-derived exosomes carry a variety of active substances and have biological functions similar to those of the source cells, mainly promoting wound healing through anti-inflammatory effects, enhancing cell proliferation, augmenting angiogenesis, and inhibiting scar hyperplasia [25-28].

MSC-derived exosomes not only promote cell proliferation and migration *in vitro* and reverse the related damage mediated by IL-1β but also inhibit the secretion of pro-inflammatory factors, such as TNF-α and IL-β, promote expression of the anti-inflammatory factors IL-10 and ARG1, and regulate the polarization of macrophages toward an M2 type [22]. The use of MSC-derived exosomes for the treatment of burns in animal models can accelerate skin wound healing and angiogenesis and increase the expression of the endothelial cell marker CD31 at the wound site [22]. Through exosome-mediated angiotensin 2 (Ang-2) transfer, expression of the Ang-2 protein in the wound area and HUVECs is upregulated, which enhances HUVEC migration and angiogenesis [22]. Therefore, MSC-derived exosomes have clear would-healing-promoting activity. Compared with MSCs, exosomes are associated with the advantages of low immunogenicity, high stability, low tumorigenicity, and easy preparation and have been applied for the treatment of various forms of skin injury [7].

Despite the widely observed biological and therapeutic effects of MSC-derived exosomes for wound healing and tissue repair, the development of exosomal pharmacological products is limited. The major obstacle is the difficulty in preserving the stability of exosomes in wound exudates. Therefore, it is necessary to use biomaterials to fix exosomes and synergistically promote wound healing. Liquid band-aid comprises a new type of band-aid mainly composed of film-forming materials, solvents, and functional substances. Biomaterials used for band film formation exert protective and regenerative effects on wounds through isolation of the external environment and moisture maintenance [29]. Liquid band-aids can also help to promote wet healing of wound tissue, which is a favorable condition for promoting wound repair [30, 31]. In addition, compared to traditional gauze band-aid, liquid band-aid has the advantages of being light, soft, non-irritating, comfortable, and aesthetic.

Chitosan is the product of the partial deacetylation of the natural polysaccharide chitin, which possesses cellular biocompatibility characteristics and functions in various processes, such as hemostasis, bacteriostasis, and wound healing promotion. Chitosan is widely applied in food, medicine, bioengineering, chemical industries, and other fields [32-34]. In this study, water-soluble chitosan, poloxamer 407, and poloxamer 188 were used as film-forming agents, whereas aminoacetic acid acted as a hemostatic agent to prepare the liquid band-aid. Based on the smoothness and film-formation speed of the liquid band-aid, the composition of the liquid band-aid was optimized as 1% chitosan solution, 15% poloxamer 407 / poloxamer 188, and 0.1% aminoethyl acid (pH 5.0–7.0). MSC-derived exosomes were encapsulated in chitosan liquid band-aid, and their activities *in vitro* and *in vivo* were verified. This is a creative study because, for the first time, we have encapsulated biologically active exosomes in a chitosan liquid band-aid. *In vitro* evidence indicates that the exosome liquid band-aid can significantly promote the proliferation and migration of HUVECs, representing an important cell type in the skin. The co-culture with chitosan exosome liquid band-aid significantly increased the number of HUVECs and promoted HUVEC wound closure after scratching. Furthermore, the antibacterial test showed that the chitosan exosome liquid band-aid had a good ability to inhibit *E. coli* and *S. aureus*. These results indicated that the chitosan liquid band-aid can inhibit bacteria and promote vascular endothelial cell proliferation and migration.

The therapeutic effect of the chitosan exosome liquid band-aid was validated using a mouse wound model. Treating full-layer skin trauma in mice with the chitosan exosome liquid band-aid resulted in no obvious changes in body weight, indicating no overt toxic side effects. The chitosan exosome liquid band-aid further promoted wound healing in mice, and the wounds healed completely 14 days after treatment. Pathological analysis revealed that the exosome liquid band-aid could also effectively reduce the inflammatory response of the wound, promote the formation of blood vessels, and facilitate wound healing. These results indicate that the chitosan exosome liquid band-aid could help sustain exosomes locally in the wound and effectively improve their effects on skin wound healing.

## CONCLUSION

In summary, for the first time, we developed a liquid band-aid with MSC-derived exosomes for wound healing. The results demonstrated that liquid band-aid, synergistically with MSC-derived exosomes, promoted wound healing in mice, thus ultimately providing a novel strategy for wound healing.

## Figures and Tables

**Table 1:** The optimized formulation of liquid band-aid.

**Concentration of Chitosan**	**Poloxamer 407/Poloxamer 188**	**Concentration of Amino Acetic Acid**	**Smoothness**	**Film-formation Speed**
0.5%	10%	0.1%	Surface smoothness: 4 Surface smoothness after rubbing: 3	Good
-	15%	0.1%	Surface smoothness: 4 Surface smoothness after rubbing: 4	Excellent
-	20%	0.1%	Surface smoothness: 4 Surface smoothness after rubbing: 4	Good
-	25%	0.1%	Surface smoothness: 4 Surface smoothness after rubbing: 5	Good
-	30%	0.1%	Incomplete dissolution	-
1.0%	10%	0.1%	Surface smoothness: 4 Surface smoothness after rubbing: 5	Good
-	15%	0.1%	Surface smoothness: 5 Surface smoothness after rubbing: 5	Excellent
-	20%	0.1%	Surface smoothness: 5 Surface smoothness after rubbing: 4	Excellent
-	25%	0.1%	Surface smoothness: 4 Surface smoothness after rubbing: 4	Good
-	30%	0.1%	Incomplete dissolution	-
2.0%	10%	0.1%	Surface smoothness: 5 Surface smoothness after rubbing: 5	Good
-	15%	0.1%	Surface smoothness: 4 Surface smoothness after rubbing: 4	Good
-	20%	0.1%	Surface smoothness: 4 Surface smoothness after rubbing: 3	Excellent
-	25%	0.1%	Incomplete dissolution	-
-	30%	0.1%	Incomplete dissolution	-
3.0%	10%	0.1%	Surface smoothness: 5 Surface smoothness after rubbing: 5	Good
-	15%	0.1%	Incomplete dissolution	-
-	20%	0.1%	Incomplete dissolution	-
-	25%	0.1%	Incomplete dissolution	-
-	30%	0.1%	Incomplete dissolution	-

## Data Availability

The data and supportive information are available within the article.

## LIST OF ABBREVIATIONS

hUC-MSCs: Human Umbilical Cord Mesenchymal Stem Cells
hUC-MSC-Exo: Mesenchymal Stem Cell-derived Exosomes
CS: Chitosan
HUVECs: Human Umbilical Vein Endothelial Cells
Con: Control
NTA: Nanoparticle Tracking Analysis
H&E: Hematoxylin-eosin
